# Psychosis risk individuals show poor fitness and discrepancies with objective and subjective measures

**DOI:** 10.1038/s41598-021-89301-5

**Published:** 2021-05-10

**Authors:** Katherine S. F. Damme, Richard P. Sloan, Matthew N. Bartels, Alara Ozsan, Luz H. Ospina, David Kimhy, Vijay A. Mittal

**Affiliations:** 1 Department of Psychology, Northwestern University, 2029 Sheridan Rd., Evanston, IL 60208 USA; 2 Institute for Innovations in Developmental Sciences (DevSci), Northwestern University, Evanston/Chicago, IL USA; 3 Department of Psychiatry, Columbia University, New York, NY USA; 4 Department of Rehabilitation Medicine, Albert Einstein College of Medicine, Bronx, NY USA; 5 Department of Rehabilitation Medicine, Montefiore Medical Center, New York, NY USA; 6 Department of Psychiatry, Northwestern University, Chicago, IL USA; 7 Department of Psychiatry, Icahn School of Medicine at Mount Sinai, New York, NY USA; 8 MIRECC, The James J. Peters VA Medical Center, Bronx, NY USA; 9 Medical Social Sciences, Northwestern University, Chicago, IL USA; 10 Institute for Policy Research (IPR), Northwestern University, Chicago, IL USA

**Keywords:** Human behaviour, Risk factors

## Abstract

Exercise is a promising intervention for individuals at clinical high-risk for psychosis (CHR). However, these youth may not be reliable reporters on fitness. There have been no investigations that utilized objective fitness assessment in this population. The present study objectively characterizes the level of fitness in CHR youth, compares the accuracy of self-report measures to objective fitness indices, and explores clinical factors that may influence the accuracy of self-reported measures of fitness. Forty CHR individuals completed an exercise survey and objective indices of fitness (i.e., VO_2_max and BMI). Forty healthy volunteers completed objective indices of fitness and a structured clinical interview ruling out the presence of psychiatric illness. CHR youth showed greater BMI and lowered VO_2_max compared to healthy volunteers. In the CHR group, self-report items (perceived fitness) did not reflect objective indices of fitness, whereas specific exercise behaviors (intensity of exercise) showed stronger correlations with objective fitness measurements. Exploratory analyses suggested that symptoms (grandiosity and avolition) related to errors in self-perception. Results indicate that CHR individuals are less fit than controls as indexed by objective measures of fitness and that it is important to consider unique population clinical characteristics when employing self-report data.

## Introduction

Exercise is a promising new area of intervention for individuals across many stages of psychosis, including those at clinical high risk for psychosis (CHR)^1–8^. Recent intervention research suggests that exercise may combat disease-driven pathophysiology, including increased hippocampal connectivity and volume^9,10^. Despite this promise, substantially less work has examined unique factors of the CHR population. CHR specific work would inform effective, early intervention by defining critical health characteristics and starting fitness levels in CHR populations^7,9,11,12^. Additionally, extant CHR research has relied solely on self-report measures of physical activity to assess fitness^9,11,13–15^, with few exceptions^9,15^, which may limit the validity of these findings. Critically, fitness self-report measures have been vulnerable to the influence of episodic memory, emotional state effects, and cognitive biases/reframing^16,17^. This issue is particularly acute in studies of individuals with or at risk for psychosis, given the substantial episodic memory deficits documented in these populations^18–20^. Additionally, symptoms implicated in distortions of reality (i.e., grandiosity) may impact perceived fitness. Similarly, deficits in motivation (i.e., avolition) may also influence perceptions of exercise rigor or exertion. Accordingly, the current study examined the relationship between multiple objective metrics of fitness and self-reported measures of fitness in individuals at CHR for psychosis. This study also examined the particular impact of symptoms on self-reported fitness. Collectively, this information can serve to refine both assessments of fitness and targets fitness intervention.

A growing body of research has demonstrated that exercise interventions are effective in improving cognitive function^1,4,5,12,21^, neurocognitive function^2,12^, and connectivity^2^ for individuals across the psychosis spectrum, including risk mental states, CHR, the first episode of psychosis, and schizophrenia populations. Existing psychosis literature has established that psychosis is associated with poorer physical health^1,6,15,21,22^, but it is unclear if individuals at CHR for psychosis have poorer health^23,24^. Extant evidence has suggested that CHR individuals are less active^9^ and self-report lower fitness levels, less physical activity, and more barriers to exercise^11–15^. These reductions in fitness may result from symptoms: negative symptoms reducing motivation for activity^25,26^, social anxiety leading to reduced social sports engagement^27^, or perceived barriers to exercise^11^. Additionally, CHR individuals engage in behaviors that may decrease fitness, such as cannabis use^28,29^ and nicotine use^28^. In contrast, several studies have suggested that this does not translate into lower levels of biometric fitness, stating instead that CHR individuals have a normative range of body mass index (BMI; 1–4). However, existing mentions of BMI have been demographic descriptions for exclusion purposes^30–33^; as a result, BMI is not well characterized in CHR individuals. Clarifying the nature and extent of fitness deficits in early risk for psychosis would provide critical insight into developing and refining targets of exercise interventions in these populations^6,7,24,34^.

In the extant literature on CHR individuals, there are a number of inconsistencies between self-reported and descriptive levels of fitness that may reflect methodological issues surrounding assessments of fitness. For example, biometric evaluations of fitness (such as actigraphy and BMI) may not capture the most relevant features of fitness for CHR. Instead, physiological measures of fitness, such as VO_2_max, may be more sensitive assessments of emerging health issues^20,23,24^. To date, only one study has examined physiological or objective measures of fitness (i.e., VO_2_max) in a genetic risk model of psychosis, individuals with psychotic-like experiences, and/or future conversion to psychosis. This study found that VO_2_max was significantly lower in these individuals compared to peers^15^. These findings may imply that physiological health metrics (i.e., VO_2_max) may be a more sensitive assessment of health than BMI^22,24,34^ for risk populations such as CHR.

Inconsistencies in the literature may also reflect issues related to a self-report approach. Indeed, self-report accounts of fitness behaviors have limited validity in capturing actual health behaviors even in healthy individuals^20,35–37^. In CHR populations, this self-report validity may be further limited by psychosis-like experiences^38^, such as distortions in self-perception. In fact, several symptoms have good face validity as confounding variables in fitness self-report. Grandiosity, for example, is an exaggerated sense of superiority/belief in special powers, which may lead to an overestimation of health and fitness. In contrast, avolition symptoms reflect an impaired motivational drive, which may distort experiences and reports around the physical effort of physical activity. As a result, individuals with high levels of avolition may experience moderate levels of exertion as more strenuous. If such distortions impact self-reported fitness, then it may be critical to assess fitness objectively in this population to accurately evaluate the potential impact of exercise as a treatment.

The current study examined if CHR individuals show reduced objective measures of fitness compared to peers. These analyses established whether CHR individuals are objectively less fit than peers. Next, self-report measures of fitness were compared to CHR individuals' objective fitness. These analyses provide critical insight into whether objective measures of fitness provided additional insight beyond self-report measures of fitness. Further analyses also investigated whether attenuated psychotic symptoms impact self-reported experiences and perception of fitness. Finally, follow-up analyses explored whether the degree of mismatch between self-reported, perceptions of physical fitness and actual fitness (i.e., participant error) may reflect specific symptoms that impact the subjective experience of physical activity due to distortions of self (i.e., grandiosity) or the impairments in motivation (i.e., avolition).

## Methods

### Participants

The group comparison analyses combined independent studies of aerobic fitness. CHR individuals’ data were collected at The Adolescent Development and Preventive Treatment (ADAPT) lab. The healthy volunteer sample was selected as the best match for the age range to the CHR sample from a larger study conducted at the New York State Psychiatric Institute (NYSPI) at the Columbia University Medical Center (CUMC). At both sites, all participants provided written informed consent. In the ADAPT lab, 40 CHR individuals completed a structured clinical interview assessing attenuated psychotic symptoms for inclusion into the study and to rule out the presence of a psychotic disorder. CHR subjects also completed an exercise survey (e.g., current exercise practices, perceived physical fitness, and objective indices of fitness, i.e., VO_2_max and BMI). Among the CHR group, six individuals were treated with SSRIs, five were treated with stimulants, one person was treated with mood stabilizers, and no subjects were treated with antipsychotics. At Columbia University, 40 healthy volunteers completed objective indices of fitness and a structured clinical interview ruling out the presence of psychiatric illness. For inclusion, healthy control subjects could not exceed American Heart Association standards for average fitness -defined as a maximum aerobic fitness (V̇o2max) ≤ 43 males and 37 females. Healthy volunteers were excluded if screening indicated symptoms of affective disorder, psychosis, or substance abuse, or current psychotropic medication. Additionally, healthy control subjects were excluded if they had any medical condition that affected the autonomic nervous system or cardiovascular system. Participants were recruited from advertising (healthy volunteers); inclusion criteria required healthy volunteers to meet American College of Sports Medicine’s standards for exercise^39^ to ensure safety during participation in the fitness assessment.

### Ethical approval statement

Northwestern University and New York State Psychiatric Institute at Columbia University Medical Center’s Institutional Review Board approved guidelines for all study procedures. All study procedures were carried out in accordance with institution-approved guidelines and regulations. All participants were above 18 years of age.

### Clinical assessment of symptoms

All subjects completed the Structured Clinical Interview for DSM-IV Axis I Disorders (SCID) to rule out any psychosis diagnosis for both the CHR and healthy volunteer groups. CHR participants completed the Structured Interview for Psychosis Risk Syndromes (SIPS) to assess the presence of a CHR syndrome and track attenuated symptoms. Exploratory analyses included positive and negative domain totals and specific symptoms. The SIPS is made up of 19 items that are rated on a scale from 0 to 6 by an interviewer. These items are grouped into larger symptom dimensions of psychosis (e.g., positive and negative). The SIPS scale contains an instrument referred to as the Scale of Prodromal Symptoms (SOPS). On the SOPS an interviewer rates the severity of symptoms along a 7-point scale ranging from absent (0) to severe and psychotic (> 6). Advanced doctoral students served as the study interviewers were trained over a 2-month period after their inter-rater reliabilities exceeded the minimum criterion of Kappa ≥ 0.80. In the current study, individuals were grouped as clinical high risk if they met the criteria for attenuated positive symptoms syndrome (i.e., recently emerging symptoms that occur at a weekly frequency or long-standing symptoms that have recently escalated).

### Self-reported fitness scale

CHR participants completed a self-report survey comprised of items from many validated measures. Subscales of this self-report have been previously reported in assessing physical activity in CHR individuals^11,13^. The items selected for the current study include Perceived Fitness, Frequency of Exercise, Time Spent Exercising, and Intensity of Exercise. Perceived fitness was rated by participants on a Likert-type scale that ranged from 0 (Poor) to 3 (Excellent). The frequency of exercise, where exercise included any activity that resulted in sweating or rapid heart rate, was rated based on a typical week and ranged from 0 (rarely or never) to 3 (five or more times). Time spent exercising was rated on a scale spanning 0 (< 30 min) to 3 (> 60 min). The intensity of exercise assessed the frequency that these exercise sessions resulted in sweating or rapid heart rate on a scale from 0 (never) to 3 (always; every time).

### Objective indices of aerobic and biometric fitness

Objective indices of fitness included VO_2_max and Body Mass Index (BMI). VO_2_max indexes an individual’s ability to transport and use oxygen at the maximum capacity during aerobic exercise. At both sites, body mass index (BMI) was calculated based on height and weight measurements collected in the lab using the U.S. Department of Health and Human Services׳ National Heart Lung and Blood Institute (http://nhlbisupport.com/bmi/bminojs.html) BMI online calculator. The error of fitness estimation was calculated by relating objective fitness metrics to self-reported perceived fitness. The standard model error was computed for each subject, reflecting the difference between the actual values of self-reported perceived fitness and the expected values of fitness according to objective fitness metrics. Individuals were then categorized into inaccurate and accurate reporter groups consistent with their quartile distribution of standardized errors. Those individuals who were inaccurate in their estimates (either highly over- or under-estimating their fitness) were grouped as inaccurate reporters; those with more typical amounts of error in their estimates (near the 50^th^ percentile of standard model error) were grouped as accurate reporters for follow-up analyses.

At Northwestern, an expert exercise physiologist conducted a modified Balke max-exercise protocol^41^ under the supervision of a physician. In this modified Balke max-exercise protocol, the treadmill speed was set to elicit 70% of the age-predicted max heart rate and an RPE rating of around 13 (“somewhat hard”). This target was achieved by maintaining the speed of the treadmill remained the same throughout the test, but adjusting the incline of the treadmill belt increased 2% every 2 min (or 2.5% for speeds 6 mph or greater). The participants’ heart rate and ratings of perceived exertion (RPE) determined the final treadmill speed. Staying within these parameters generally yields an 8–12 min test. During the protocol, the speed of the treadmill remained the same, but the incline of the treadmill belt increased 2% every 2 min (or 2.5% for speeds 6 mph or greater). Tests generally lasted 8–12 min, the recommended target for VO_2_max testing^42^.

The Human Performance Laboratory at Columbia University Medical Center assessed the VO_2_max of participants during cycling. The tests were performed on an electronically-braked cycle ergometer that was calibrated prior to every test (Ergoline 800S electronic-braked cycle ergometer (SensorMedics Corp., Anaheim, CA). Participants completed a four-stage protocol: 1) 5-min resting baseline, 2) 3-min no-resistance warm-up, 3) increasing speed and resistance until 10–15 W with maximum exercise norms (total duration of approximately 12-min), 4) active recovery of 3-min. During the test, the workload was increased 10–15 W every 1-min until one of four exercise criteria was reached. To generate the VO_2_max (mL/kg/min) variable, participants achieved one of the following four criteria during exercise: VO_2_ plateau, 85% of maximal heart rate (220-age), respiratory quotient ≥ 1.1, or self-reported exhaustion as indexed by the Borg Scale^43^ to generate the VO_2_max (mL/kg/min) (MAX-1, PHYSIO-DYNE Instrument Corp., Quogue, NY) . For more detail on the full exercise procedure, refer to Kimhy et al., 2014.

Exercise methods, gender, and age-appropriate z-scores were created for all VO_2_max scores separately for each exercise type (treadmill and cycling) according to the American Heart Association^44^ national norms to ensure comparability across sites. By using national normalization guidelines, we aim to reduce the impact of local site features- local fitness opportunities and habits- as well as account for the difference in exercise intervention type (i.e., treadmill and cycling), which are expected to elicit distinct levels of VO_2_max. This approach is consistent with recommendations put forth in extant population normalization aerobic fitness measures^45^.

### Analytical approach

Participant demographic comparisons across groups were conducted on sample features using chi-square analyses to characterize categorical sample features and t-tests to examine the continuous features of the sample. Group analyses were designed to contextualize the objective fitness to self-reported fitness perception and are treated as exploratory. For multiple comparisons, the group comparisons (CHR vs. healthy volunteers) will be treated as independent analyses from the within CHR group analyses. As a result, the within-group (CHR only) analyses will be corrected for the two comparisons (objective fitness to self-report fitness; objective fitness to symptoms), and findings were treated as significant if the *p* < 0.025, a Bonferroni correction. Any follow-up analyses with particular symptoms will be interpreted cautiously and treated as exploratory. For all significant analyses, follow-up analyses will be conducted to examine if sex or age contributes significantly to the models. Variables that significantly contribute to the model or impact the direction or magnitude of the reported findings will be included as a nuisance variable, in line with Miller and Chapman (2001)^46^.

Separate simultaneous general linear models compared groups (i.e., CHR and healthy volunteers) on objective metrics of health, where group membership predicted fitness (VO_2_max or BMI, respectively). Analyses within the CHR group examined whether self-report indices of fitness related to objective indices; to limit the total number of analyses, self-report subscales were entered simultaneously into separate general linear models to predict VO_2_max and BMI in separate analyses. This approach has the added benefit of accounting for multiple self-reported features of exercise. Any significant subscale provided added insight into the relevance of particular items over and above other items.

Similarly, a repeated-measure general linear model examined the relationship between clinical symptoms (positive and negative) as the within-subject measure. The subscales of self-reported indices of fitness were entered simultaneously as the between-subjects measure in a single model to reduce the number of total comparisons. In this approach, a significant relationship to symptoms would indicate that the self-reported indices of fitness related to symptom severity, and interaction by scale would indicate that the relationship varies by symptom type (positive or negative). Finally, in a set of exploratory analyses, CHR individuals were grouped by error quintiles as accurate or inaccurate (based on the relationship of their perceived fitness to each objective fitness) and were compared to symptoms of grandiosity and avolition in separate t-tests: grandiosity by VO_2_max accuracy quintile, grandiosity by BMI accuracy quintile, avolition by VO_2_max accuracy quintile, avolition by BMI accuracy quintile.

## Results

### Participants

Our sample included 80 participants (healthy volunteers = 40, CHR = 40; see Table 1). There were no significant differences in sex by group, χ^2^ = 0.46, *p* = 0.49. Although the groups used distinct exercise methods (i.e., treadmill, cycling), the peak heart rate was not significantly different, *t*(78) = 0.13, *p* = 0.89, across CHR (*M* = 181.61 *StD* = 17.93) and healthy volunteers (*M* = 181.13 *StD* = 11.73). The similarity of peak heart rate suggests the exercise was of comparable rigor. There was a significant difference in age between groups, *t*(78) = -9.19, *p* < 0.001*,* where CHR individuals were younger (*M* = 20.95, *StD* = 1.38) than healthy volunteers (*M* = 23.98, *StD* = 1.56)*.* In line with Miller and Chapman, all group comparisons will include age in the model as a nuisance covariate.

**Table 1:** Group demographics and objective exercise metrics.

Variables	CHR	Control	
	Mean (StD)	Mean (StD)	Statistics
Age (years)	20.95 (1.37)	23.98 (1.56)	*t*(78) = − 9.19, *p* < .001
Sex (% female)	40.0%	47.5%	χ2 = 0.46, p = .49
BMI (kg/m^2^)	24.10 (5.76)	22.55 (2.85)	*F*(1,77) = 12.29, *p* = .001
AHA corrected VO_2_Max	− 0.97 (0.178)	− 0.38 (0.178)	*F*(1,77) = 4.01, *p* = .049
Positive symptom total	12.84 (3.8)	–	–
Negative symptom total	7.42 (5.7)	–	–
Grandiosity symptom score	1.95 (1,3)	–	–
Avolition symptom score	1.66 (1.6)	–	–
Peak heart rate	181.61 (17.93)	181.13 (11.73)	*t*(78) = 0.131, *p* = .89
Resting heart rate	88.6 (18.60)	81.5 (10.50)	*F*(1,79) = 4.42, *p* = .039
Current smoker	1/40	0/40	–
Current cannabis use	6/40	–	–
Ethnicity (% Latinx)	4.25%		–
**Race**			
Asian	7.5%		
Black	15%		
Central/South American	15%		
White (European)	22%		
Interracial	7.5%		
**Income**			
> $10,000	2%		
$10–19,999	20%		
$20–39,999	20%		
$40–59,000	9%		
$60–99,999	20%		
> $100,000	18%		
Do not know or prefer not to answer	11%		

### Group differences objective indices of fitness

**Figure 1:**
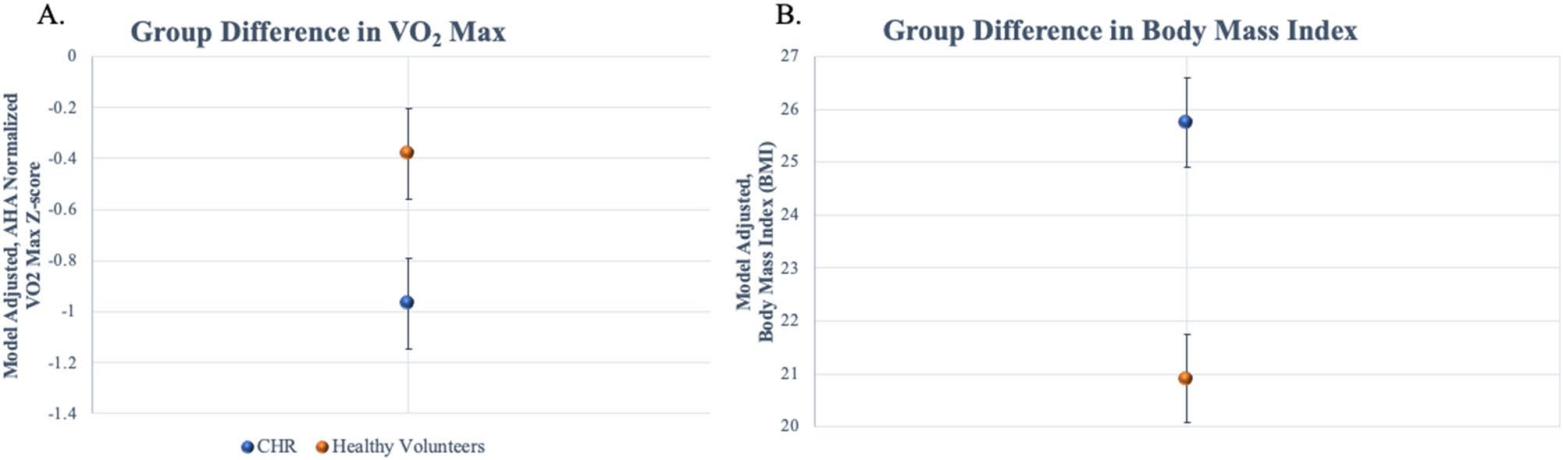
CHR physical markers of fitness and perceived fitness metrics: CHR group differences in VO_2_ max **(A)** and body mass index **(B)**.

In a general linear model, group (CHR vs. Healthy Volunteer) related to normed VO_2_max (ml/kg/min) z-scores accounting for variability related to age as a nuisance variable. There was a significant main effect of group, *F*(1,77) = 4.01, *p* = 0.049, such that the CHR group (*M* = -0.97, *StD* = 0.178) had significantly lower VO_2_max compared to healthy volunteers (*M* = -0.38, *StD* = 0.178), indicating reduced physiological health, Fig. 1a. There was also a significant main effect of age, *F*(1,77) = 5.99, *p* = 0.017, such that older age was related to lower VO_2_max values, *r*_*-partial*_ = − 0.27. In a general linear model, group (CHR vs. Healthy Volunteers) related to BMI (kg/m^2^), accounting for variability related to age. There was a significant main effect of group, *F*(1,77) = 12.29, *p* = 0.001, such that the CHR group (*M* = 25.75, *StD* = 0.84) had significantly increased BMI compared to healthy volunteers (*M* = 20.91, *StD* = 0.84), indicating reduced health, Fig. 1b. There was also a significant main effect of age, *F*(1,77) = 12.29, *p* = 0.001.

### CHR self-reported fitness items related to objective indices of fitness

**Figure 2:**
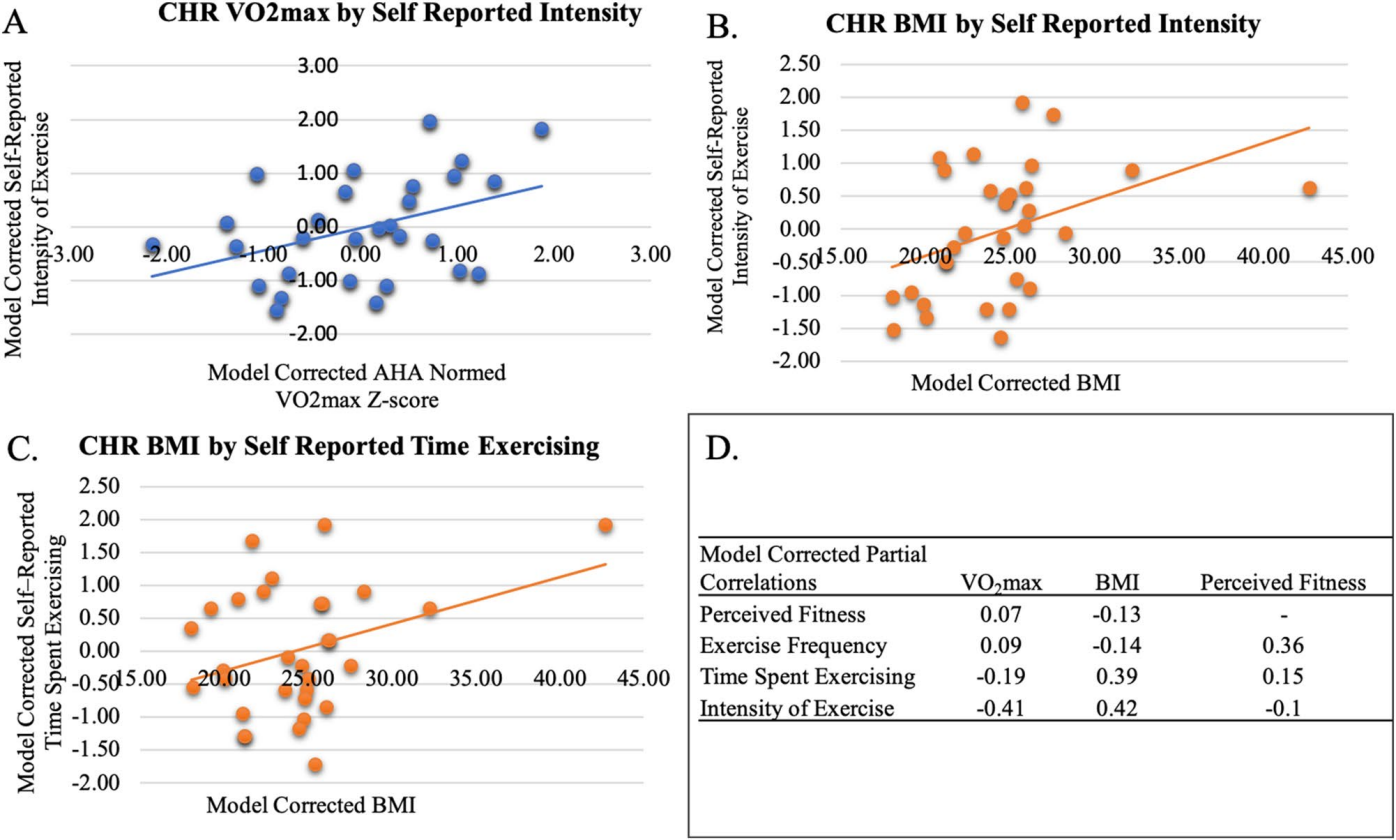
Relationship of objective physiological health to self-reported measures of fitness: **(A)** Within the CHR group relationships between VO_2_ max to reported exercise intensity, **(B)** BMI to reported exercise intensity, **(C)** BMI to reported time spent exercising, **(D)** intercorrelation matrix of the perceived and actual fitness metrics.

In a general linear model, self-reported fitness items (Exercise Frequency, Time Spent Exercising, Intensity of Exercise, Perceived Fitness) were entered simultaneously to predict VO_2_max (ml/kg/min) z-scores. Perceived Fitness did not predict VO_2_max values (*p* = 0.72) but did relate to the self-reported intensity of the exercise item, *F*(1, 36) = 5.17, *p* = 0.03, *r*_*partial*_ = − 0.42, Fig. 2a. There were no other specific exercise features related to VO_2_max values, *p*’s > 0.21. In a general linear model, self-reported fitness subscales (Exercise Frequency, Time Spent Exercising, Intensity of Exercise, Perceived Fitness) were entered simultaneously to predict BMI. Perceived Fitness did not predict BMI values (*p* = 0.47) but the self-reported intensity of the exercise item, *F*(1, 36) = 5.79, *p* = 0.02, *r*_*partial*_ = 0.42, Fig. 2b, and the self-reported time spent exercising item, *F*(1, 36) = 4.97, *p* = 0.03, *r*_*partial*_ = 0.39, Fig. 2c, each predicted BMI. BMI did not relate to the self-reported frequency of exercise item, *p* = 0.47, summarized in Fig. 2d.

### CHR self-reported fitness related to symptoms

In a repeated-measures general linear model, attenuated psychotic symptoms (positive and negative symptom scales) were related to self-reported fitness subscales (Exercise Frequency, Time Spent Exercising, Intensity of Exercise, Perceived Fitness), accounting for sex. Perceived fitness related to symptoms, even when accounting for specific self-reported fitness behaviors, *F*(1, 34) = 5.90, *p* = 0.02. Additionally, follow-up analyses demonstrated that sex significantly contributed to the overall model, *F*(1, 34) = 4.26, *p* = 0.04, with CHR males subjects showing more severe symptoms (model-corrected mean = 11.02, *SEM* = 1.05) compared to CHR females (model-corrected mean = 7.94, *SEM* = 0.84). Age was also examined as a potential contributor to the model but did not significantly contribute to the model, nor did it change the magnitude or direction of the reported effects, *p* = 0.78.

### CHR errors in perceived fitness related to symptoms

**Figure 3:**
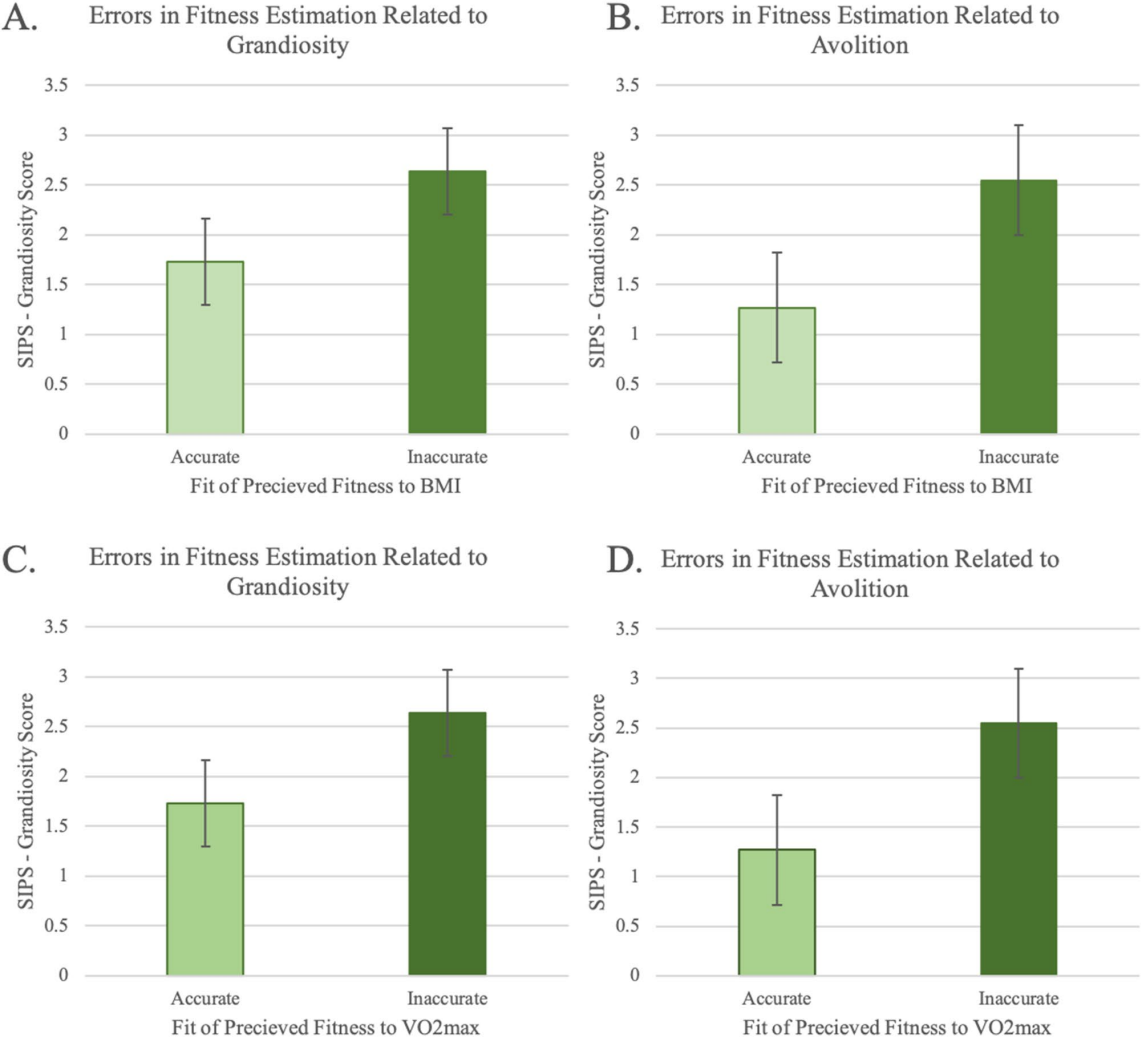
Errors in perception related quartiles groups (inaccurate and accurate) for BMI (top panels) and VO_2_max (bottom panels) to symptoms of **(A,C)** grandiosity and **(B,D)** Avolition.

In separate t-tests, BMI related to errors in perception (how far the actual fitness deviated from the predicted fitness) for both grandiosity, *t*(38) = 2.28, *p* = 0.04, Fig. 3a, and avolition, *t*(38) = 2.55, *p* = 0.02 Fig. 3b. VO_2_ max related to errors in perception (how far the actual fitness deviated from the predicted fitness) for both grandiosity *t*(38) = 2.29, *p* = 0.04, Fig. 3c, and avolition, *t*(38) = 2.55, *p* = 0.02, Fig. 3d. Follow-up analyses were conducted examining positive and negative symptom totals to examine specificity, which did not relate to errors in perception (how far the actual fitness deviated from the predicted fitness) *p*’s > 0.12. Follow-up analyses examined the potential contribution of age and sex to the models above and found that they did not contribute to the model significantly, *p*’s > 0.51.

## Discussion

The current paper was the first to take a comprehensive assessment of fitness in CHR individuals. CHR individuals were significantly less fit than peers in terms of objective physiological (VO_2_max; Fletcher et al., 2001) and biometric (BMI) measures of fitness. Self-reported perceptions of fitness^11,13,20^ did not reflect either objective metric of fitness (VO_2_max, BMI), but objective items regarding fitness behaviors (i.e., intensity and time spent exercising) related to the objective metrics of fitness. Finally, errors in perceived fitness^38^ were related to a distorted perception of self (i.e., grandiosity) and motivation (i.e., avolition).

CHR individuals showed lower levels of fitness on objective physiological (VO_2_ max;^1,41,44^ and biometric (BMI) measures in separate analyses, compared to healthy volunteers. This lower objective fitness emphasized the potential benefit of exercise as an early, non-invasive intervention in CHR individuals^6,7,9^. This finding extended the psychosis literature, suggesting that a psychosis spectrum diagnosis is associated with lower fitness^1,6,15,21,22^ including individuals with attenuated psychotic symptoms. Taken together, the emergence of attenuated symptoms co-occurred with a decline in physical fitness may have reflected an overall deterioration in neurological fitness. This deterioration of symptoms might have led to a decline in both engagement in fitness activities and health^1,2,6^. These findings emphasized the importance of objective metrics of fitness^38^. Future studies should evaluate fitness longitudinally as these markers of health may confer additional risk for conversion and may be a therapeutic target for individuals at greatest risk.

Self-report perceived level of fitness^13^ did not reflect objective metrics of fitness overall^1,40,41,44^. Self-reported intensity of exercise^13^ did reflect physiological fitness as expected; increased self-reported exercise intensity related to an increased VO_2_max capacity^1,41^. In contrast, self-reported time spent exercising and intensity of exercise^13^ corresponded to an increased BMI. This inconsistency with BMI highlights may be a limitation of BMI; BMI does not distinguish between the content of body mass in terms of whether the weight reflects increased muscle mass or body fat^47–49^. Individuals with high BMI may have reflected a heterogeneous fitness group comprised of individuals with high body fat percentage and elevated weight due to muscle mass^47,49^. Alternatively, self-reported intensity and time spent exercising may reflect the individual’s subjective experience^38,43,50^. Individuals with a higher BMI may experience more difficulty exercising and report objectively less intense exercise as more intense and as lasting a longer period of time. In addition to biases of subjective experience, self-report of fitness behaviors may be further distorted by the presence of attenuated symptoms^38,50^.

Self-reports of perceived fitness^13^ reflected the overall severity of positive and negative symptoms, even when accounting for other features of health behavior (e.g., frequency and intensity of exercise). Exploratory analyses modeled the difference in perceived fitness to actual fitness; the errors of perceived fitness were related to specific symptoms of self-perception (i.e., grandiosity) and motivation (i.e., avolition). Higher symptoms of grandiosity and avolition are related to more distortion in self-perception of fitness. Errors in self-perception of fitness were defined as the discrepancy between self-reported perceived fitness and the scores that we would predict based on objective indices of fitness. These findings build on extant findings of clinical influence on some self-report measures^38,51–54^. Collectively these symptom analyses support the possibility that symptomatology may distort the ability of CHR individuals to accurately self-assess fitness. Future studies should also examine the possibility that these errors may be affected by deficits in memory.

Despite the many strengths and novelty of this study, there are some relevant limitations. First, the healthy controls did not complete the same self-reported fitness measure, as a result it is unclear if these distortions in self-reported fitness are unique to the CHR group or a larger problem with self-reported fitness data^20,35–37^. It is notable, however, that self-report items did relate to the symptoms that uniquely define individuals at CHR, which somewhat mitigates concerns regarding the relevance of these self-reported discrepancies to features of the CHR group. Although BMI is a useful metric in distinguishing CHR from healthy controls, it was not entirely clear if increased BMI reflected increased muscle mass or body fat percentage^47–49,55^. Future studies on this topic should consider including additional metrics of fitness such as waist to hip ratio^22^ or skinfold thickness^55^ to estimate body fat percentage in addition to body mass index. It is also notable that the current group comparisons were exploratory analyses of available samples and were less than ideal comparisons. Future work should collect both CHR and healthy control subjects from the same location and complete the same exercise protocol, which should be repeated at least once. Additionally, future studies should match groups on critical features, such as BMI, age, sex, race, and ethnicity. Finally, future studies should also include questionnaires that are more common to the larger exercise literature, including the Simple Physical Activity Questionnaire (SIMPAQ;^56^ this would help integrate future findings into a larger exercise literature.

The current paper attempted to address these potential differences from the two contributing sites varied in the specific exercise approach (e.g., treadmill and cycling) by normalizing the data using a national population standard^44,57^. Although this correction is not perfect, the concern about site differences may be somewhat mitigated by the equivalent peak heart rates across exercise types, which suggests that the exercises were of roughly equivalent rigor. Finally, the sites varied according to age; although the model accounted for variability related to age, it remains possible that age impacts the current findings. It is notable that the difference in age between samples was quite small (3 years); as such, the current study is not well suited to interpret the impact of age on fitness.

The current study sample was similar in size to comparable extant literature^1,9,11,32^. Nevertheless, the field would benefit from larger sample sizes to examine additional variables that may affect fitness, including local access to exercise, culture, race, income, and ethnicity. Additionally, this smaller sample size restricted our sensitivity to symptoms within the CHR group. As a result, analyses were restricted to strong candidate subscales. Notably, the presence of significant relationships, despite this reduced power, suggests that this area is a promising line of inquiry for future studies. However, these follow-up analyses should be interpreted with appropriate caution, given their exploratory nature.

In conclusion, despite CHR individuals being objectively less fit than their peers, their self-reported perceived fitness related to symptoms and not objective levels of fitness. Additionally, the errors in perceived fitness may reflect grandiosity and avolition. These findings add to a larger literature that suggests attenuated positive and negative symptoms contribute to a disturbed perception of self^54^. Future studies should use caution in depending on self-report measures of fitness alone. Additionally, exercise interventions may benefit from emphasizing objective self-report items (e.g., time spent exercising) and objective metrics (VO_2_max) or concrete features of exercise behavior (time and intensity of exercise) rather than relying on subject recall^38^ or subjective intensity^50^.
